# Effects of Build Direction on the Mechanical Properties of a Martensitic Stainless Steel Fabricated by Selective Laser Melting

**DOI:** 10.3390/ma13225142

**Published:** 2020-11-15

**Authors:** Ling-Chieh Shen, Xi-Huai Yang, Jeng-Rong Ho, Pi-Cheng Tung, Chih-Kuang Lin

**Affiliations:** Department of Mechanical Engineering, National Central University, Jhong-Li District, Tao-Yuan City 32001, Taiwan; cherry6314559@gmail.com (L.-C.S.); gjacky7205@gmail.com (X.-H.Y.); jrho@ncu.edu.tw (J.-R.H.); t331166@ncu.edu.tw (P.-C.T.)

**Keywords:** selective laser melting, martensitic stainless steel, build direction, mechanical properties, anisotropy, residual stress

## Abstract

Mechanical properties and microstructure are investigated for a martensitic stainless steel (AISI 420) fabricated by selective laser melting (SLM) in three build directions. The tensile specimens built by SLM are classified into three groups. Group A is horizontally built in the thickness direction, Group B is horizontally built in the width direction, and Group C is vertically built in the length direction. The loading direction in tensile test is parallel to the build direction of Group C, but perpendicular to that of Groups A and B. Experimental results indicate build direction has significant effects on the residual stress, hardness, and tensile properties of SLM builds. Microstructural analyses indicate the as-fabricated SLM AISI 420 builds exhibit elongated cells and acicular structures which are composed of martensite and retained austenite phases growing along the build direction. Such anisotropy in the microstructure leads to anisotropic mechanical properties as Group C specimens (length direction) exhibit greater yield stress, ultimate tensile stress, and elongation than the specimens of Groups A (thickness direction) and B (width direction). The residual compressive stress in the gauge section also contributes to the superior tensile properties of Group C (length direction), as compared to Groups A (thickness direction) and B (width direction), which exhibit residual tensile stress in the gauge section.

## 1. Introduction

Additive manufacturing (AM) has recently been applied for direct fabrication of near net shape components. It is different from the conventional manufacturing processes which cut or remove material to make the final shape, while it adds material during the fabrication process [1]. Metallic AM techniques can reduce the consumption of material and have flexibility on fabricating components with three-dimensional (3D) complex structures that could not be done by conventional manufacturing processes [2]. Several AM techniques have been developed for building metallic parts, including selective laser melting (SLM), electron beam melting (EBM), laser engineered net shaping (LENS), and binder jetting (BJG) [3]. A high power energy beam is employed to locally melt the feedstock of powder or wire material and join materials layer by layer in the metallic AM techniques [1]. Among the well-known metallic AM techniques, SLM is a type of powder bed fusion process in which a laser beam is used to selectively fuse a metallic powder bed in a layer-by-layer manner [3]. Due to a repeated process of melting and solidification, epitaxial grain growth tends to develop along the building direction in the metallic AM parts [1]. As a result, metallic AM builds tend to have anisotropic and heterogeneous microstructures and textures due to process-induced rapid directional solidification, leading to anisotropic and heterogeneous mechanical properties [4].

Effects of build direction on the microstructure and mechanical properties have been studied for several metallic alloys produced through SLM processes, such as Ni-based superalloys [5,6,7,8], titanium alloys [5,9,10,11,12,13,14], aluminum alloys [5,15,16], and steels [5,17,18,19,20,21]. Chen et al. [5] reveal that metallic AM parts of various materials exhibit layer-wise morphologies in the cross sections along the build direction, leading to an anisotropic microstructure. Deng et al. [6] investigated the build direction effect on the mechanical properties of an Inconel 718 superalloy fabricated by SLM and found that the horizontally built parts show higher tensile strength but lower ductility than the vertically built ones. The anisotropy in tensile behavior is mainly attributed to the different amounts of residual stress and dislocations accumulated in the built parts [6]. In another study on SLM built Inconel 718 superalloy by Du et al. [7], the yield strength and ultimate tensile strength increase with an increase in the incline angle of build direction from 0° to 45°. The dependence of microstructure and tensile behavior on build direction is related to the temperature gradient during deposition and the resulting anisotropy in the crystal orientation [7]. Tensile properties of a TC21 titanium alloy built by SLM in two directions show significant anisotropy, as reported in the study of Zhang et al. [9]. The horizontally built specimens demonstrate better tensile strength but inferior elongation, compared to the vertically built ones [9]. The anisotropic tensile properties of SLM TC21 are attributed to a microstructural variation of α phase features in different build directions [9]. The effects of build direction on tensile and fatigue performance of a Ti6Al4V titanium alloy built by SLM in three orientations (0°, 45°, and 90°) were studied by Sun et al. [14]. Build direction is found to have a significant effect on fatigue performance but a slight influence on tensile strength and ductility of the SLM built Ti6Al4V alloy [14]. The 45° built samples exhibit better tensile and fatigue properties than the 0° and 90° built samples due to a smaller number of process defects in the builds [14]. An SLM built A357 aluminum alloy exhibits a greater yield strength and elongation in the horizontal build direction than that in the vertical build direction as a result of the formation of a crystallographic texture [16].

For a 316L stainless steel produced through SLM, horizontally built specimens exhibit the highest fatigue resistance followed by vertically built ones, while diagonally built specimens possess the lowest fatigue strength, as presented by Shrestha et al. [18]. Such anisotropy in the fatigue behavior of SLM 316L stainless steel is induced by the variation in layer orientation and directionality of process defects with respect to the loading direction [18]. As found in another study on SLM 316L stainless steel by Liverani et al. [19], tensile strength and fatigue resistance is decreased with a change of build direction from 45° to 90°, while elongation is increased. No build direction effect on density is observed in the study of Liverani et al. [19]. For an SLM fabricated 300 maraging steel (18Ni), considerable anisotropy of tensile behavior is found in the as-built conditions in which horizontally built specimens possess the highest tensile strength and ductility [20]. On the other hand, the vertically built specimens have the lowest tensile strength, while the diagonally built ones have the poorest elongation [20]. However, in the study of Tan et al. [21] on another 300 maraging steel built by SLM in two directions, anisotropy of hardness and tensile properties is barely observed and a slight build direction effect is only found on the impact fracture behavior. Apparently, the build direction effect on the mechanical properties of metallic AM parts differs from one material to another. This is attributed to the variations in microstructure, type and directionality of process defect, and loading direction [4].

AISI 420 is a martensitic stainless steel which has excellent mechanical properties and moderate corrosion resistance. It is used in a wide range of applications from low to high temperatures, including injection molds, pressure vessels, steam generators, surgical instruments, blades, and others [22]. Recently, a conformal cooling channel has been employed in the plastic injection mold because it can provide a more uniform temperature distribution, shorter cycle time, and better product quality. However, a conformal cooling channel cannot be manufactured by conventional machining techniques due to a complicated design of channel geometry inside the mold. The AM process is then introduced to fabricate such a mold for which AISI 420 is favorably considered as the mold material due to the low cost and high absorption of laser radiation [23]. However, only limited work in the literature has been focused on the characteristics of AISI 420 fabricated by AM processes. An SLM process window of laser power and scanning speed is established for AISI 420 powder to reach a relative density over 99% by Zhao et al. [23]. The AISI 420 parts built by such an SLM process have a high hardness of 50.7 HRC, which is applicable to the plastic injection mold [23]. Saeidi et al. [24] demonstrate that a heat treatment conducted at 400 °C on the as-built SLM AISI 420 can improve the tensile yield strength and ductility, making it an ultra-high strength stainless steel with high ductility. As described above, the previous findings indicate the importance of build direction on microstructure and performance of metallic SLM builds. However, the effect of build direction on the microstructure and mechanical properties of AISI 420 fabricated via SLM has not yet been systematically studied. As the material’s multiaxial mechanical properties are needed for design of mechanical components fabricated by the SLM technique, the aim of this study is to investigate the relationship between build direction and mechanical properties of SLM built AISI 420 parts. The specimens investigated in this study are fabricated by an SLM process in three build directions. A mechanical test is performed to obtain tensile properties. Microstructure, density, hardness, and residual stress are also analyzed for the built parts. It is hoped that the results in this study can provide useful information on improving the quality of SLM built AISI 420 parts for employment in the plastic injection mold [23] and other applications.

## 2. Experimental Procedures

### 2.1. Specimen Fabrication

**Figure 1:**
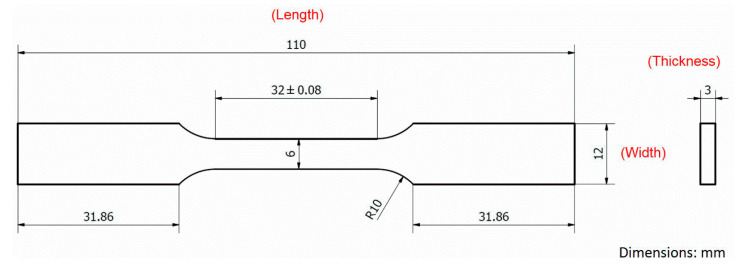
Geometry and dimensions of tensile specimen.

**Figure 2:**
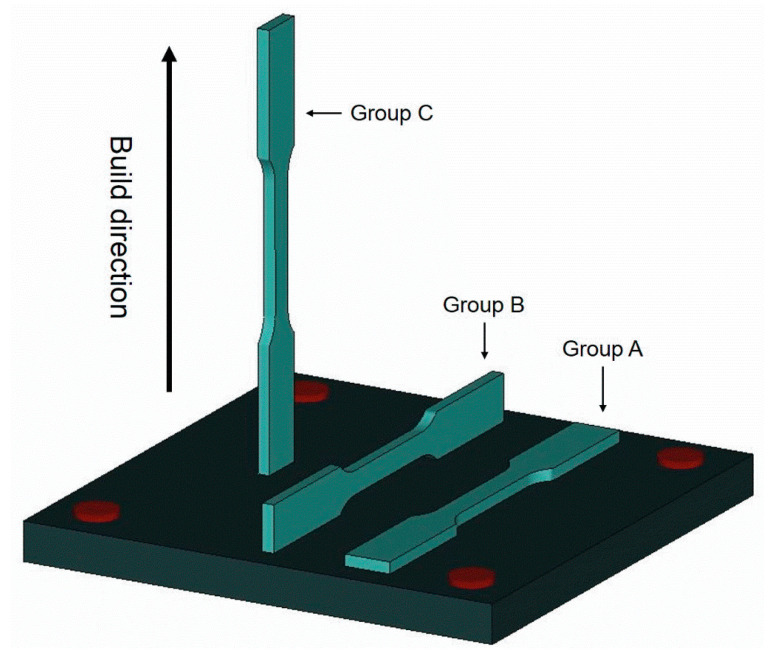
Schematic of build direction for each group of specimen.

Commercial AISI 420 stainless steel powder (Sandvik Osprey Ltd., Neath, UK) is used to fabricate the tensile test specimens. According to the data sheet provided by the vendor, the powder size is in the range of 10–53 μm and the major elements in chemical composition include 12.0–14.0 wt% Cr, 0.3–0.4 wt% C, ≤ 1.0 wt% Si, ≤ 1.0 wt% Mn, ≤ 0.6 wt% Ni, and balance of Fe. Specimens are fabricated by a commercial powder-bed SLM machine (OPM250L, Sodick Corporation, Ltd., Kanagawa, Japan). The SLM machine is equipped with an ytterbium-doped fiber laser, a nitrogen gas generator, a powder supply mechanism, and a working table. The given SLM process parameters and scanning strategy are listed in Table 1. An AISI 420 stainless steel baseplate is used and preheated to 180 °C. Laser power is set as 420 W for the first three layers to ensure a robust bonding between the built part and baseplate. After that, laser power is reduced to 400 W until the final top layer is finished. Figure 1 shows the geometry and dimensions of the tensile test specimen. The tensile specimens are classified as Groups A–C based on the build direction. As shown in Figure 2, specimens in Groups A–C are built along the thickness direction (*t*), width direction (*w*), and length direction (*l*), respectively. In other words, Group C is vertically built, and Groups A and B are horizontally built in the short transverse and wide transverse directions, respectively. Three specimens are fabricated for each group. After the SLM process, the built specimens are cut out of the baseplate by wire electrical discharge machining. To investigate the inherent effects of build direction on the microstructure, residual stress state, and mechanical properties, the specimens are kept in the as-built condition. Therefore, heat treatment is not applied to the specimens.

### 2.2. Measurement of Density, Hardness, and Residual Stress

**Figure 3:**
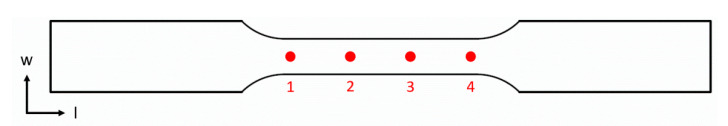
Positions selected for residual stress measurement.

Densification of the SLM specimens is measured by Archimedes’ principle. The accuracy of the electronic balance for measuring the mass of density sample is 0.01 g. The relative density of the SLM specimen is defined as *ρ*_r_ = *ρ*_sample_/*ρ*_reference_. *ρ*_sample_ is the density of the specimen and *ρ*_reference_ = 7800 kg/m^3^ is the reference density of AISI 420 steel [25]. A hardness tester (AR-10, Akashi Corporation, Japan) is used to measure the hardness in Rockwell C scale (HRC) by applying a load of 150 N. The accuracy of the hardness tester is 0.1 HRC. Each sample is indented at ten different places in the grip section. The residual stresses at selected positions are measured using a portable X-ray residual stress analyzer equipped with CrK*_α_* radiation (μ-X360s, Pulstec Industrial Corporation, Ltd., Shizuoka, Japan). An X-ray beam of 2-mm diameter is employed to determine the residual stress, using a cos α method and two-dimensional detectors (imaging plates). Note that the error range of each measurement is about ± 25 MPa. Details of such residual stress measurement method are given in Reference [26]. Four positions on the centerline in the gauge section (Figure 3) are selected for residual stress measurement in each SLM tensile specimen before tensile test. As shown in Figure 3, both the normal stress components *σ_l_* and *σ_w_* in the length (*l*) and width (*w*) directions, respectively, are measured at each selected position.

### 2.3. Uniaxial Tensile Test

To determine the tensile properties, such as yield stress, ultimate tensile stress, and elongation, uniaxial tensile test is conducted using a commercial servo-hydraulic material test machine (MTS 810, MTS System Corporation, Eden Prairie, MN, USA). The accuracy of the load cell for measuring the applied load is 1 N. A uniaxial extensometer (MTS 634.12F-24, MTS System Corporation, Eden Prairie, MN, USA) is attached on the gauge section to measure the strain during testing. The mechanical test is performed under displacement control with an initial stroke rate of 0.5 mm/min followed by 2 mm/min when the strain reaches 1%. An engineering stress–strain curve is then obtained for each specimen tested.

### 2.4. Fractography and Microstructural Analysis

After tensile testing, fracture surface is observed using a scanning electron microscope (SEM, S-800, Hitachi Ltd., Tokyo, Japan) to find the fracture origin and fracture pattern. An attached energy dispersive spectrometer (EDS) is used to determine the elemental composition at specific regions. Optical microscopy (OM, BX51M, Olympus Corporation, Tokyo, Japan) and SEM in backscattered electron (BSE) mode are employed to investigate the microstructure. To explore the grain growth direction, some cross sections and surface areas are selected for microstructural observation. These metallographic samples are ground using sandpapers up to #2000 and polished using 1-μm and 0.3-μm Al_2_O_3_ paste. After polishing, the samples are chemically etched for 20–30 s in an acidic solution of 2% HF and 8% HNO_3_. For better imaging results, the SEM specimens are sputter coated with a thin conductive Pt film. In addition, X-ray diffraction analysis (XRD) is applied to determine the crystalline phases in the given SLM specimens. The XRD samples are taken out of the gauge section. The X-ray energy applied is 15 KeV with a wavelength of 0.82656 Å. The beam size is 500 μm and the diffraction signals during measurement are recorded using an image plate detector (mar345s, marXperts Corporation, Norderstedt, Germany). The X-ray diffraction data are refined using the General Structure Analysis System (Los Alamos National Laboratory, CA, USA) to obtain the *θ*-2θ X-ray diffraction profiles.

## 3. Results and Discussion

### 3.1. Density and Hardness

The relative density measured for each sample is listed in Table 2 and it ranges from 90% to 98%. Note that in Table 2 and Table 3 (Section 3.3), A1, A2, and others individually represent each specimen ID number. In general, Group A has the highest average relative density (96%), followed by Group B (93%) and then Group C (92%). Note that Group A has the smallest height of the SLM build while Group C has the largest one. Therefore, the given SLM process parameters lead to a slightly higher density in the builds with a smaller height. However, the density of the SLM builds in this study is lower than that of other as-built SLM AISI 420 parts which have a relative density greater than 99% [23,24]. Note that the build direction is not given in References [23,24] and the largest dimension in their SLM builds is only 40 mm which is much shorter than the 110 mm given in this study. This might partially explain why our relative density is somewhat lower than theirs. Table 2 also lists the hardness (HRC) data measured at ten different places for each specimen, as represented by the average value ± standard deviation. The hardness of the given specimens in as-built state is higher than that of the counterparts made by conventional manufacturing techniques of which the hardness is about 50 HRC [23]. For similar as-built SLM AISI 420 investigated in other studies, a hardness of HRC less than 51 is reported in References [23,27] while an Hv 650 (equivalent to HRC 63) is reported in reference [24]. Therefore, the average hardness of each given build direction in this study is greater than that in reference [23,27] but lower than that in reference [24]. As shown in Table 2, Group C generally has the greatest hardness, followed by Group A and then Group B. This is consistent with the trend that Group C specimens also possess the highest tensile strength among the given three build directions, as described below. Apparently, build direction indeed has an effect on the hardness of the given SLM AISI 420 builds. More discussion on such build direction effect is given in Section 3.5.

### 3.2. Residual Stress

**Figure 4:**
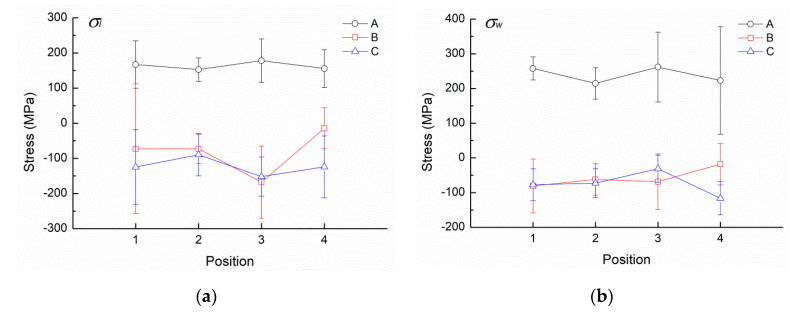
Results of residual stress measurement: (**a**) *σ_l_*; (**b**) *σ_w_*. (A, B, and C denote the specimens built along the thickness, width, and length directions, respectively, as shown in Figure 2).

Residual stresses are unavoidably generated in SLM builds due to high temperature gradient and high cooling rate caused by the local heat input of high-energy laser beam during the SLM process [3]. The residual stress may influence the strength of SLM builds depending on the residual stress level and loading direction [3]. Residual stress measurement results for the as-built SLM specimens are shown in Figure 4 in which the symbol represents the average value and the attached error bar represents the standard deviation. Note that the average value and the standard deviation of each data point presented in Figure 4 are calculated based on all of the repeated measurements of three samples in each given build direction. As shown in Figure 4, the standard deviation is somehow large at certain measured positions, which may be attributed to the variation of surface roughness from one sample to another. Surface roughness has certain effects on the measurement of residual stress using the given X-ray diffraction method [28]. As the tensile specimens in this study are directly fabricated by SLM and tested in the as-built state without surface polishing, it is expected that the unpolished, rough surface may cause a certain extent of scattering on the measurements of residual stress. Despite the data scattering, a comparison of the residual stress distribution among the given build directions can still be made using the general trend of the average values presented in Figure 4. For Group A specimens, residual tensile stresses exist in both *l*- and *w*-directions on the top length-width plane. This is expected because the measured points of Group A are on the final layer in the SLM build. The process of cooling and contraction of the melt material after being heated by the laser generates the tension seen in the deposited layer [29]. Accordingly, the most recently added layers will experience the highest level of tension, while eventually the lower layers are subject to compression by the cooling and contraction of the melt material added above [29]. Group B and C specimens exhibit nearly zero stress or residual compressive stress in a similar trend at the measured points, as these measured points are located on the side surface in the middle section of the SLM build.

### 3.3. Tensile Properties

**Figure 5:**
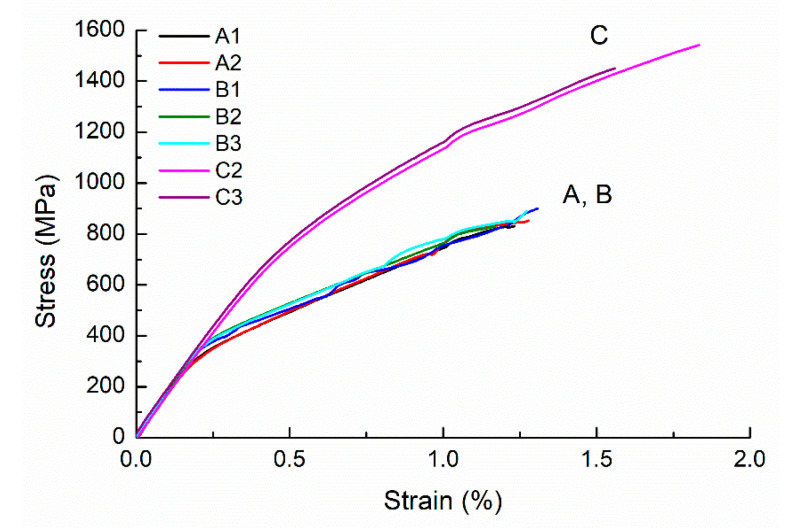
Stress–strain curves of SLM AISI 420 built in three directions. (A, B, and C denote the specimens built along the thickness, width, and length directions, respectively, as shown in Figure 2. A1, A2, and others individually represent each specimen ID number).

Representative stress–strain curves of specimens tested are shown in Figure 5. The 0.2% offset method is employed to determine the yield stress in Figure 5. As no necking phenomenon occurs, the ultimate tensile stress is the same as the fracture stress. Elongation is defined as the final strain value when the specimen fractures. The stress–strain curves indicate that the as-built SLM AISI 420 stainless steel is a brittle material as its elongation is less than 2% regardless of build direction. A similar phenomenon is also observed for another as-built SLM AISI 420 [24]. Tensile properties obtained for each specimen are listed in Table 3. In comparison of yield stress, ultimate tensile stress, and elongation, Group C is superior to Groups A and B. The yield stress of Group C is almost twice that of Groups A and B. Compared to the as-built SLM AISI 420 reported in a previous study by Saeidi et al. [24], Group C has a comparable ultimate tensile strength but a higher yield strength. However, the elongation to fracture observed in this study (1.17–1.83%) is smaller than that (3.5 ± 0.3%) observed in reference [24]. It might be attributed to a lower relative density of the as-built specimens in the present study. The anisotropy of tensile properties observed in current work is caused by the difference in build direction as a directional heat transfer is established along the build direction in SLM fabricated metals [4]. Consequently, a directional grain growth mechanism would cause elongated grains to form along the build direction [4]. The mechanism responsible for such anisotropic phenomena in the present study will be discussed in Section 3.4.

### 3.4. Fractography and Microstructural Analysis

**Figure 6:**
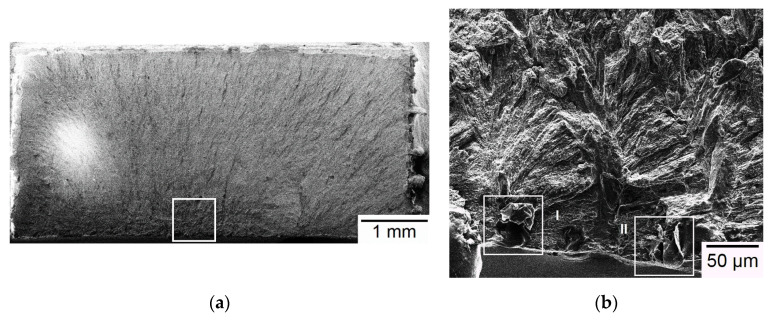

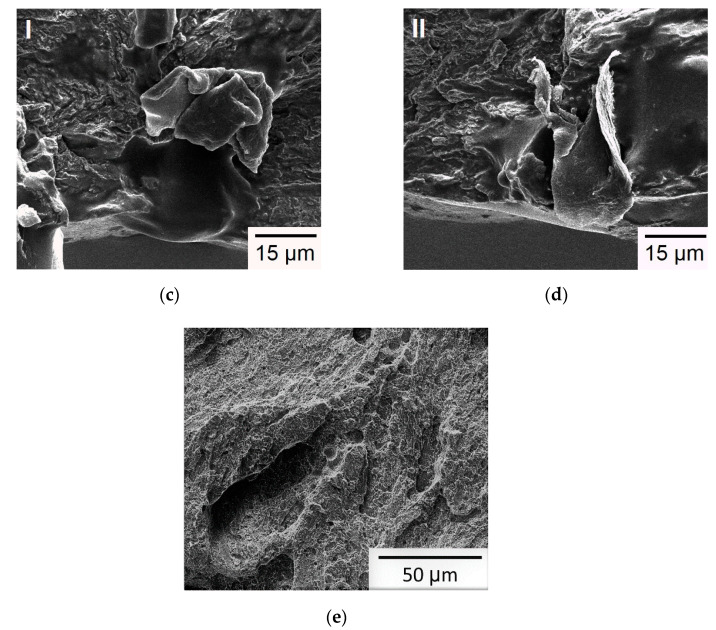
SEM micrographs of a Group A specimen: (**a**) Overview of fracture surface; (**b**) high-magnification view of the outlined fracture origin zone in (**a**); (**c**) defect I in (**b**); (**d**) defect II in (**b**); (**e**) high-magnification view of a region away from the fracture origin.

Typical SEM micrographs of fracture surface of Group A specimens are shown in Figure 6. Figure 6a shows a typical flat, brittle fracture mode without any ductile fracture feature such as necking or dimples. The fracture origin is located at the width edge as outlined by the square zone in Figure 6a. Two apparently defect-associated origins are found at the edge of the square zone in high-magnification view, as shown in Figure 6b–d. EDS is employed to analyze the difference in composition between these two defects and other regular, non-defect regions. It reveals these defects contain a small amount of iron (0–16 wt%) and chromium (0–3 wt%) and a large amount of carbon (61–71 wt%), compared to other non-defect regions. Therefore, these two defects are considered inclusions formed during the SLM process. In addition, it is noticed that the width edge at which fracture initiates is also located on the built top layer of the Group A specimen. As described in Section 3.2, residual tensile stress exists on the top length-width plane (final deposited layer) of the Group A specimen. As a result, fracture initiates at the existing inclusions in the residual-tensile-stress region of the as-built SLM specimen. As shown in Figure 6e, a typical region away from the fracture origin also exhibits planar facets of brittle fracture without ductile fracture feature of dimples. In this regard, brittle fracture is the dominating failure mechanism for Group A specimens.

**Figure 7:**
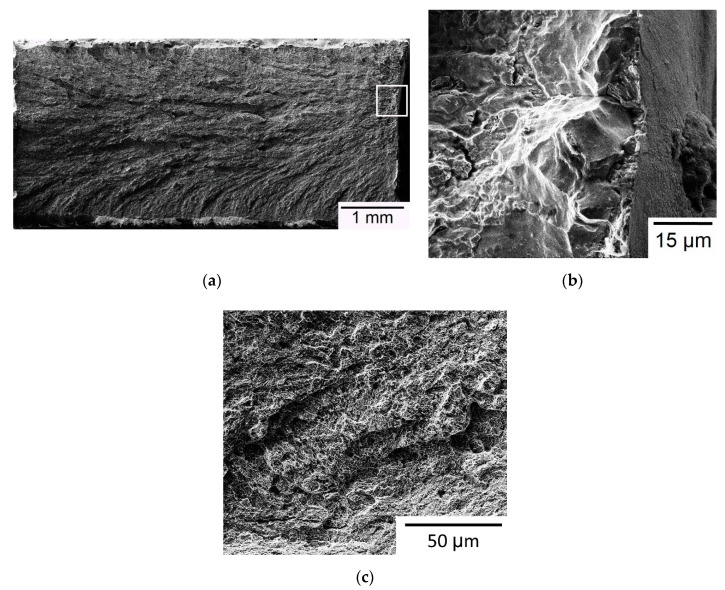
SEM micrographs of a Group B specimen: (**a**) Overview of fracture surface; (**b**) high-magnification view of the outlined fracture origin zone in (**a**); (**c**) high-magnification view of a region away from the fracture origin.

A representative fracture surface of Group B specimens is shown in Figure 7. Again, a typical flat, brittle fracture pattern is found and evidenced by the planar facets shown in the high-magnification micrographs of Figure 7b,c. For this specimen, fracture initiates at the thickness edge, as indicated by the outlined square region in Figure 7a. No obvious inclusion is found in the fracture origin region, as shown in the high-magnification micrograph of Figure 7b. EDS is also applied to analyze the composition of fracture origin and other regular regions. The results indicate the fracture origin in this Group B specimen is not an inclusion because it contains a high proportion of iron. The thickness edge at which fracture originates is located on the top built layer in the gauge section of the Group B specimen. Group B specimens are built along the width direction such that the final top layer in the gauge section is on the length-thickness plane. Residual tensile stress is also expected to exist on this top layer of the Group B specimen promoting fracture initiation. Note that the residual stress measurements shown in Figure 4 for Group B are taken on the vertical side surface rather than on the top surface of the SLM build. Apparently, residual tensile stress also plays an important role in determining the fracture initiation site.

**Figure 8:**
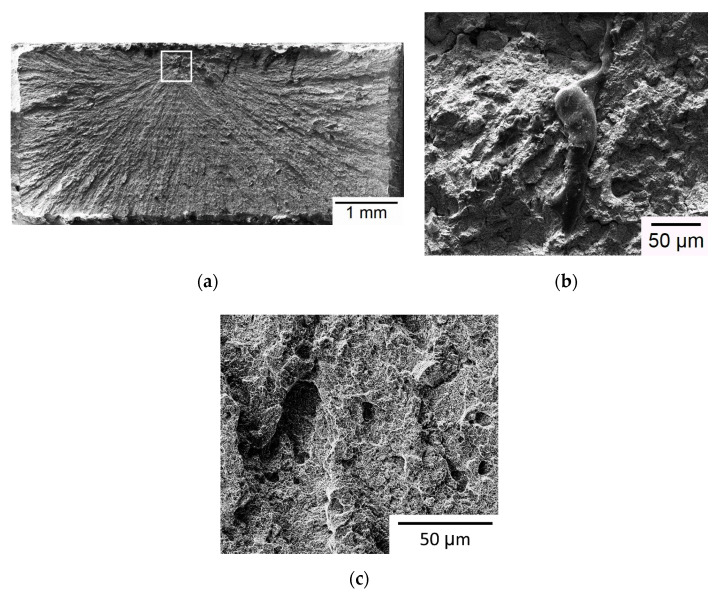
SEM micrographs of a Group C specimen: (**a**) Overview of fracture surface; (**b**) high-magnification view of the outlined fracture origin zone in (**a**); (**c**) high-magnification view of a region away from the fracture origin.

**Figure 9:**
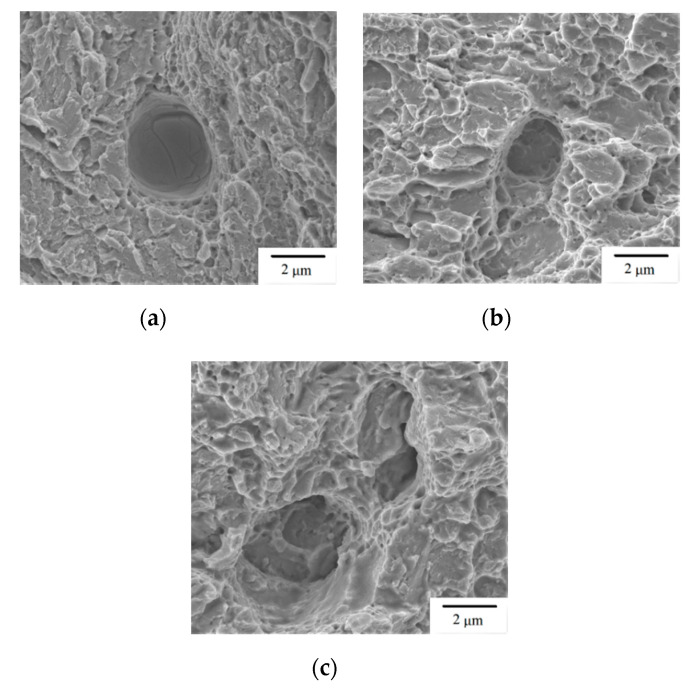
SEM micrographs of micro-pores and cleavage facets observed on the fracture surface: (**a**) Group A; (**b**) Group B; (**c**) Group C.

Figure 8 shows the typical fracture surface of Group C specimens. Figure 8a also shows a flat, brittle fracture surface, and fracture origin is identified at a location inside the outlined square zone. Both the fracture origin zone (Figure 8b) and other region (Figure 8c) exhibit a brittle fracture pattern of planar facets. The fracture origin in this case is not located at the edge of gauge section, which is different from the presented cases of Groups A and B. Note that the top layer of SLM build in Group C is located at the grip section rather than at the gauge section. As shown in Figure 4, the residual stress (*σ_l_*) along the loading direction is compressive in the gauge section for the Group C specimen. Comparison of EDS analysis between the fracture origin and other regions indicates the fracture origin is also an inclusion containing a large amount of carbon (73 wt%) and a small amount of iron (10 wt%) and chromium (1 wt%). Crack tends to initiate at such irregular inclusion due to a greater stress concentration effect. Typical micro-pores observed on the fracture surfaces of the given SLM specimens are shown in Figure 9. Such micro-pores are much smaller than the inclusions observed in Figure 6 and Figure 8. Note that planar cleavage facets are also clearly seen in the SEM micrographs of Figure 9, again, providing evidence of failure in brittle manner. In summary, inclusion and residual tensile stress are the two major factors in determining the fracture origin site for the SLM builds presented in this study. Crack tends to start from an irregular inclusion due to stress concentration and from the region with high residual tensile stress. Brittle fracture is the dominating failure mechanism in tensile test for the given SLM builds regardless of build direction.

**Figure 10:**
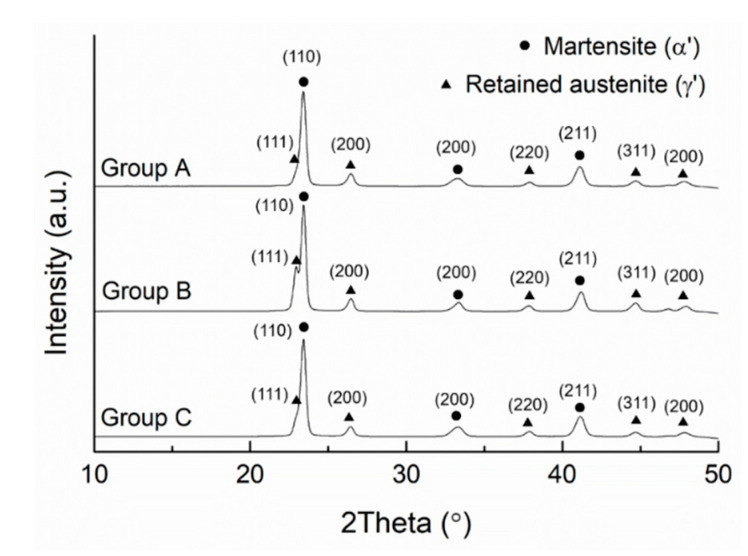
XRD results of SLM AISI 420 specimens built in various directions.

XRD results (Figure 10) indicate that martensite and retained austenite are the two major phases existing in all of the given SLM specimens. As expected, no presence of primary carbides is detected due to insufficient time for diffusion of alloying elements and formation of carbide during rapid solidification in the SLM process [30]. In addition, the repeated remelting of the material during the SLM process may cause complete dissolution of the primary carbides [30]. With a very high cooling rate in the given SLM process, the post-solidification state is similar to an austenitizing-quenching treatment for martensitic stainless steels. During the quick cooling period, a large amount of austenite transforms into martensite from the martensite-start temperature (*M_s_*) to martensite-finish temperature (*M_f_*) [31]. Some austenite retains after the martensitic transformation process is completed. As described in Section 3.2, the given AISI 420 stainless steel fabricated by SLM exhibits a brittle fracture manner with an elongation less than 2%. The reason is that the as-built SLM specimens, without any tempering treatment applied, contain a lot of fine martensite phase, so they are a brittle material. The volume fraction of retained austenite can be determined through the equation derived by van Dijk et al. [32] using the XRD results presented in Figure 10. The calculated volume fraction of retained austenite with balance of martensite is presented in Table 4 for each group of specimen. As shown in Table 4, all groups contain a lot of martensite, ranging from 71.7% to 78.8%. The amount of martensite in the given specimens is comparable to that of another as-built SLM AISI 420 steel in the study of Krakhmalev et al. [30]. Group A possesses the highest martensitic content, followed by Groups C and B. However, their difference in martensitic content is not significant, compared to the extent of difference in tensile strength. As described in Section 3.2, the tensile strength of Group C is significantly higher than that of Groups B and A. Therefore, the content of martensite may not be the controlling factor for the difference in the tensile strength of the given SLM builds.

**Figure 11:**
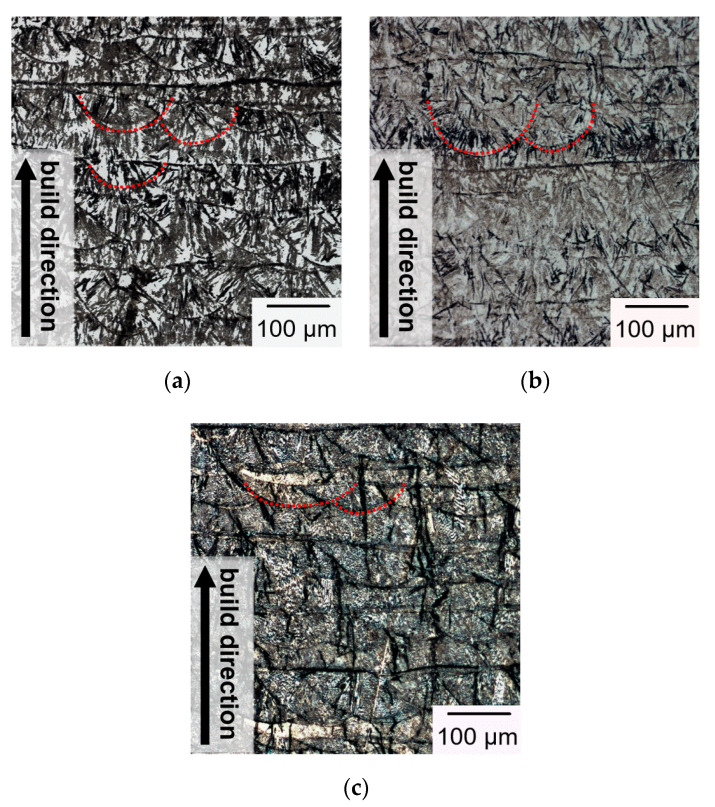
Optical microscopy (OM) micrographs of representative microstructure in the SLM specimens: (**a**) Group A; (**b**) Group B; (**c**) Group C. (Arrow indicates the build direction.).

Typical microstructure observed in low-magnification OM micrograph for each group is shown in Figure 11. Note the observed vertical cross-sections in Figure 11 are parallel to the build direction which is indicated by the arrow. Typical layer morphology is observed for each specimen presented in Figure 11 as the SLM builds are fabricated in a layer-by-layer manner. As shown in Figure 11, the melt pools exhibit semi-elliptical fusion boundaries (marked by red dash lines) on the observed planes. The top width of the melt pool is close to the focus diameter of laser spot (200 μm), while the melt-pool depth is reflective of the nominal powder layer thickness (~50 μm). As shown in Figure 11, the cross-sectional OM micrographs along the build direction exhibit similar morphologies in all of the given samples with various build directions.

**Figure 12:**
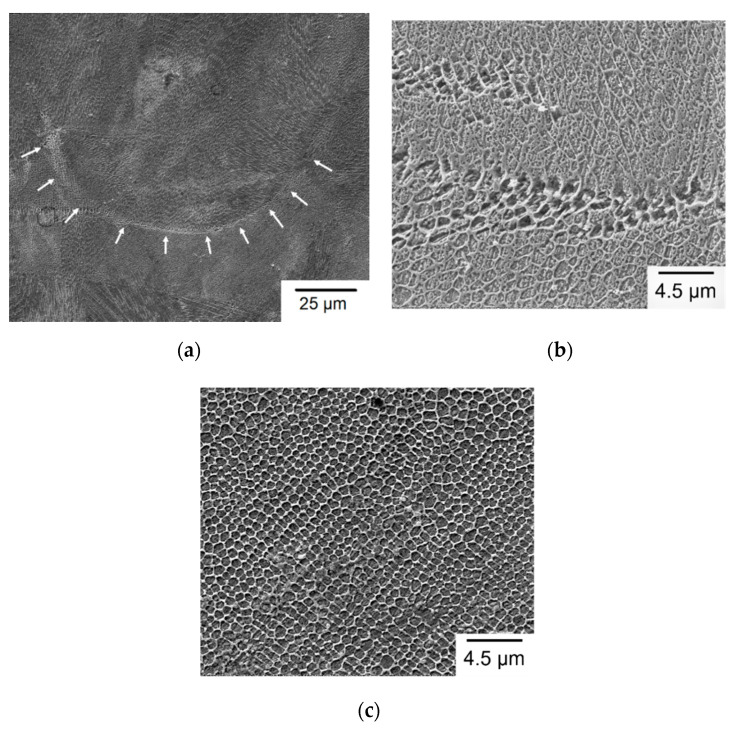
Representative SEM micrographs of melt pool: (**a**) Low-magnification view of vertical cross-section; (**b**) high-magnification view of melt pool boundary in vertical cross-section; (**c**) high-magnification view of melt pool center in horizontal cross-section.

**Figure 13:**
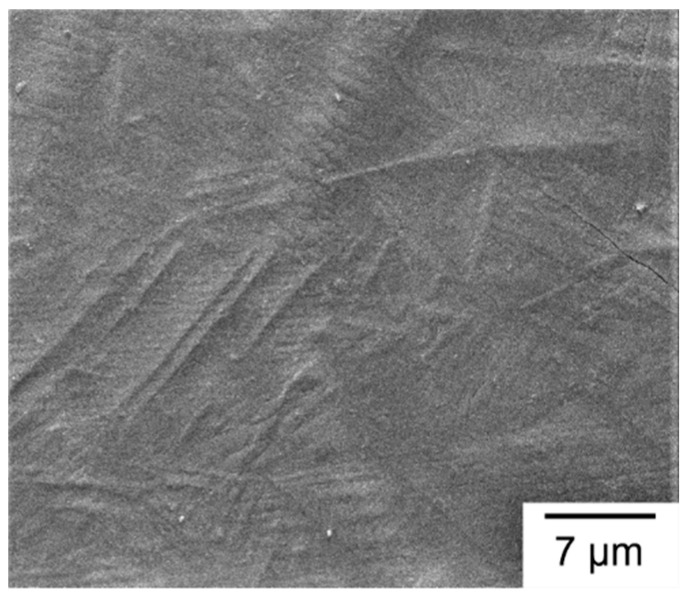
SEM micrograph of needle-like martensite laths at the top surface of as-built SLM 420 sample.

As shown in Figure 12, SEM micrographs of a Group A sample are presented as an example to display the representative melt pool microstructure for all of the given build directions. Similar to Figure 11, Figure 12a,b is taken from the vertical cross-section which is parallel to the build direction, while Figure 12c is taken from the horizontal cross-section which is perpendicular to the build direction. Figure 12a shows the low-magnification overview of a melt pool and surrounding area in which the melt pool boundary is indicated by the arrows. As shown in Figure 12a, heterogeneous microstructures including fine cells, elongated cells, and acicular structures are observed in the melt pools. Figure 12b is a high-magnification view of the area near melt pool boundary, displaying fine, elongated sub-grains. As shown in Figure 12c, fine cellular structures in the melt pool center are visible when viewed from the top. Such variation of microstructural morphology is attributed to the differences of temperature gradient and grain growth rate in various zones of a melt pool [21]. The solidification direction is along the direction having the largest temperature gradient, usually from the boundary to the center of the melt pool. Therefore, during initial solidification of a melt pool, austenite heterogeneously nucleates at the boundary and then grows toward the center to form an austenite-phase melt pool [33]. After cooling below the *M_s_* temperature, the prior austenite phase transforms to martensite during the rapid cooling stage. As a result, the cellular structures are outlined by the prior austenite grain boundaries, and the martensite laths are embedded in the sub-grain structures, as shown in Figure 12b. As shown in Figure 12b, only the retained austenite is clearly observed in the cellular structures, but the martensite laths are barely seen. The reason is that the very fine lath structure of martensite is sensitive to etching such that it is barely seen in the etched sample. In another way, SEM micrograph of the top-layer microstructure without polishing or etching is shown in Figure 13. Needle-like martensite laths in random orientation are clearly seen in Figure 13.

As shown in the OM micrographs of Figure 11, all the given SLM specimens exhibit similar microstructural characteristics in a vertical cross-sectional view along the build direction. In particular, the heterogeneous fine cells, elongated cells, and acicular structures are clearly seen in the high-magnification SEM micrographs of Figure 12a,b. In general, the elongated cellular structure grows epitaxially along the build direction. On the other hand, in the horizontal cross-sectional view of the SLM build shown in Figure 12c, fine cells are observed on the metallographic plane which is the transverse cross-section of the elongated structure. Comparison of the micrographs in Figure 12b,c clearly indicates an anisotropic microstructure in the SLM AISI 420 steel. In particular, sizes and shapes of the prior austenite grain boundary (Figure 12) are not uniform revealing a heterogeneous microstructure in the as-built SLM AISI 420 parts. It is attributed to different thermal histories in various zones of an SLM build, caused by a complex combination of several factors, including high cooling rate of melt pool, high and non-uniform thermal gradients, continuous remelting/heating of material beneath the melt pool, and overlapping of scanning tracks during the SLM process [20,24].

### 3.5. Effect of Build Direction

As described above, build direction has noticeable effects on mechanical properties, residual stress distribution, and fracture origin site. To quantitatively describe the build direction effect on mechanical properties, an anisotropy ratio is defined as *M_A_*/*M_C_* and *M_B_*/*M_C_* for the SLM builds of Groups A and B, respectively. *M_A_*, *M_B_*, and *M_C_* represent the average value of each mechanical property (yield stress, ultimate tensile stress, elongation, or hardness) of Groups A, B, and C, respectively. In other words, the mechanical properties of Groups A and B are normalized by that of Group C to define the anisotropy ratio. The calculated anisotropy ratios of various mechanical properties are summarized in Table 5. Note that the mechanical properties of Group C are taken as the basis such that its anisotropy ratios are all equal to 1 in Table 5. Table 5 indicates yield stress and ultimate tensile stress have the greatest anisotropy among the mechanical properties investigated, as the tensile strength of the horizontally built specimens (Groups A and B) is only about 50% of that of the vertically built ones (Group C). Noticeable anisotropy is also found in the elongation. However, the reduction in tensile strength of the horizontally built parts does not increase their ductility, as they exhibit even poorer elongation, with a level of only 74% of that in the vertically built ones. Compared to tensile strength and elongation, a less but still noticeable anisotropy is found in hardness and the vertically built specimens exhibit greater hardness over the horizontally ones by an extent of about 10%.

As described in Section 3.2, build direction also influences residual stress distribution in the gauge section of the tensile specimens fabricated by SLM. Residual tensile stress exists on one side (final layer) of the rectangular gauge section in Group A and B specimens, while the rectangular gauge section (without final layer) in the Group C specimens exhibits residual compressive stress. When the SLM build is subject to uniaxial tensile loading, residual tensile stress along the loading direction promotes development of cracking and fracture. This is evidenced by the fractography analysis presented in Section 3.4, indicating that fracture initiates right at the residual-tensile-stress side of the gauge section of Group A and B specimens. In other words, the fracture origins are located at the final layer of the two horizontally built groups of SLM specimens. However, for the vertically built Group C specimens, the final top layer is located at the end of grip section and the fracture initiates at microstructural defects within the gauge section rather than at the edge of gauge section. Apparently, stress concentration effect causes crack initiation at irregular defects for Group C specimens. Therefore, the residual tensile stress existing in the gauge section may cause premature fracture of the horizontally built specimens (Groups A and B) leading to a lower tensile strength and elongation, compared to the vertically built ones (Group C).

The anisotropy in mechanical properties of the given SLM builds is mainly attributed to an anisotropic microstructure, particularly the prior austenite grain boundary, as a result of directional grain growth. As evidenced by the microstructural analysis presented in Section 3.4, the orientation of the elongated cellular structures in the vertically built specimens (Group C) is parallel to the mechanical loading direction. In contrast, the orientation of the elongated cellular structures in the horizontally built specimens (Groups A and B) is perpendicular to the mechanical loading direction. Elongated columnar grains will provide a different extent of grain boundary strengthening in longitudinal versus transverse loading direction [8]. Apparently, a greater strengthening mechanism exists in the vertically built specimens (Group C) when subjected to tensile loading in the build direction. It has also been reported that more plastic deformation before failure is generally observed in the vertically orientated samples fabricated by AM [34]. Consequently, yield stress, ultimate tensile stress, and elongation of Group C are greater than those of Groups B and A. In summary, both residual stress and anisotropic microstructure contribute to the anisotropy of mechanical properties in the given SLM builds of AISI 420. Compared to the hardness and the tensile properties of other as-built SLM AISI 420 steel [23,24,27] described in Section 3.1 and Section 3.3, Group C can provide comparable and reliable mechanical properties for application in plastic injection mold.

## 4. Conclusions

(1) The dependence of microstructure and mechanical properties on build direction is confirmed for the martensitic mold steel, AISI 420 stainless steel, fabricated by SLM in a vertical direction and two horizontal directions.
(2) Build direction effect causes anisotropic mechanical properties as the vertically built SLM specimens possess superior mechanical properties (yield stress, ultimate tensile stress, elongation, and hardness) to those of horizontally built ones. This is mainly attributed to the anisotropic microstructure in which the orientation of elongated cells and acicular structures in the vertical and horizontal builds is respectively parallel and perpendicular to the tensile loading direction. The residual compressive stress existing in the gauge section also contributes to the superior tensile properties of the vertical builds, as compared to the horizontal builds which exhibit residual tensile stress in the gauge section.
(3) The SLM AISI 420 builds in as-built state exhibit elongated cells and acicular structures which are composed of martensite and retained austenite phases. The elongated cellular structures are generally oriented with the build direction due to a directional grain growth mechanism. However, build direction has a limited effect on the phase content as the SLM builds contain a comparable amount of martensite, namely 78.82%, 71.69%, and 76.71% for Groups A–C, respectively.
(4) Fractography analysis reveals residual tensile stress and irregular inclusions both play an important role in determining the fracture origin site of the given SLM builds when subjected to uniaxial tensile loading.

## Figures and Tables

**Table 1:** Selective laser melting (SLM) process parameters given in fabricating tensile test specimens.

Laser Power	420 W (First Three Layers); 400 W
Laser scanning speed	0.7 m/s
Laser spot size	0.2 mm
Layer thickness	50 μm
Baseplate preheated temperature	180 °C
Build direction	3 types (Figure 2)
Scanning strategy	Island pattern with alternating path
Hatch distance	0.2 mm

**Table 2:** Density and hardness of SLM AISI 420 specimens of various build directions.

Specimen ID	Relative Density, ρ_r_	Hardness (HRC)
A1	0.93	55.6 ± 2.4
A2	0.98	56.1 ± 2.6
A3	0.98	56.2 ± 0.6
Group A (average)	0.96	56.0
B1	0.93	49.2 ± 4.2
B2	0.94	57.3 ± 3.6
B3	0.93	52.7 ± 4.6
Group B (average)	0.93	53.1
C1	0.93	60.3 ± 4.6
C2	0.90	58.1 ± 6.0
C3	0.92	63.2 ± 4.1
Group C (average)	0.92	60.5

**Table 3:** Tensile properties of SLM AISI 420 specimens with various build directions.

Specimen ID	Yield Stress	Ultimate Tensile Stress	Elongation
	(MPa)	(MPa)	(%)
A1	481.1	830.5	1.23
A2	487.0	852.4	1.28
B1	489.9	899.3	1.35
B2	518.4	829.8	1.17
B3	511.2	890.1	1.27
C2	982.2	1541	1.83
C3	1005	1450	1.56

**Table 4:** Phase content in SLM AISI 420 specimens with various build directions.

Specimen Group	Retained Austenite	Martensite
	(%)	(%)
A	21.2	78.8
B	28.3	71.7
C	23.3	76.7

**Table 5:** Summary of anisotropy in mechanical properties with various build directions.

Specimen Group	Anisotropy Ratio in Yield Stress	Anisotropy Ratio in Ultimate Tensile Stress	Anisotropy Ratio in Elongation	Anisotropy Ratio in Hardness
A	0.48	0.56	0.74	0.92
B	0.50	0.58	0.74	0.88
C	1	1	1	1
